# Supplementary material for: Modeling glioblastoma heterogeneity as a dynamic network of cell states
Source: Mol Syst Biol. 2021 Sep 16;17(9):e10105. doi: 10.15252/msb.202010105 (PMC8444284; doi:10.15252/msb.202010105)
Supplement: Supplementary file 6 — Source Data for Figure 5 [file MSB-17-e10105-s004.zip › Figure5A_sourcedata/GSEA_3071/hallmarks_stateV.GseaPreranked.1623742546329/HALLMARK_ANGIOGENESIS.html]

Details for gene set HALLMARK\_ANGIOGENESIS[GSEA]

|  || Dataset | state43071 |
| Phenotype | NoPhenotypeAvailable |
| Upregulated in class | na\_neg |
| GeneSet | HALLMARK\_ANGIOGENESIS |
| Enrichment Score (ES) | -0.67700243 |
| Normalized Enrichment Score (NES) | -2.5800056 |
| Nominal p-value | 0.0 |
| FDR q-value | 0.0 |
| FWER p-Value | 0.0 |
Table: GSEA Results Summary

  

Fig 1: Enrichment plot: HALLMARK\_ANGIOGENESIS      
 Profile of the Running ES Score & Positions of GeneSet Members on the Rank Ordered List

  

| PROBE | GENE SYMBOL | GENE\_TITLE | RANK IN GENE LIST | RANK METRIC SCORE | RUNNING ES | CORE ENRICHMENT || 1 | STC1 |  |  | 433 | 0.920 | -0.0417 | No |
| 2 | APP |  |  | 570 | 0.818 | -0.0317 | No |
| 3 | FGFR1 |  |  | 862 | 0.673 | -0.0573 | No |
| 4 | VEGFA |  |  | 926 | 0.649 | -0.0408 | No |
| 5 | TNFRSF21 |  |  | 1166 | 0.573 | -0.0610 | No |
| 6 | VAV2 |  |  | 1528 | 0.475 | -0.1086 | No |
| 7 | PTK2 |  |  | 2794 | 0.290 | -0.3357 | No |
| 8 | CCND2 |  |  | 2876 | 0.282 | -0.3386 | No |
| 9 | ITGAV |  |  | 4207 | -0.442 | -0.5714 | No |
| 10 | FSTL1 |  |  | 4262 | -0.460 | -0.5614 | No |
| 11 | VCAN |  |  | 4491 | -0.537 | -0.5811 | No |
| 12 | PDGFA |  |  | 4998 | -0.929 | -0.6363 | Yes |
| 13 | COL5A2 |  |  | 5112 | -1.189 | -0.6056 | Yes |
| 14 | TIMP1 |  |  | 5134 | -1.251 | -0.5548 | Yes |
| 15 | JAG2 |  |  | 5151 | -1.321 | -0.4999 | Yes |
| 16 | NRP1 |  |  | 5211 | -1.609 | -0.4406 | Yes |
| 17 | S100A4 |  |  | 5238 | -1.817 | -0.3659 | Yes |
| 18 | SPP1 |  |  | 5272 | -2.446 | -0.2650 | Yes |
| 19 | LUM |  |  | 5277 | -2.507 | -0.1559 | Yes |
| 20 | COL3A1 |  |  | 5292 | -3.630 | 0.0006 | Yes |
Table: GSEA details [plain text format]

  

Fig 2: HALLMARK\_ANGIOGENESIS: Random ES distribution      
 Gene set null distribution of ES for **HALLMARK\_ANGIOGENESIS**
